# Investigating the Impacts of Real-Time Weather Conditions on Freeway Crash Severity: A Bayesian Spatial Analysis

**DOI:** 10.3390/ijerph17082768

**Published:** 2020-04-17

**Authors:** Qiang Zeng, Wei Hao, Jaeyoung Lee, Feng Chen

**Affiliations:** 1 School of Civil Engineering and Transportation, South China University of Technology, Guangzhou 510641, China; zengqiang@scut.edu.cn; 2 Jiangsu Province Collaborative Innovation Center of Modern Urban Traffic Technologies, Nanjing 211189, China; 3 School of Traffic and Transportation, Changsha University of Science and Technology, Changsha 410114, China; haowei@csust.edu.cn; 4 School of Traffic and Transportation Engineering, Central South University, Changsha 410075, China; jaeyoung@knights.ucf.edu; 5 Key Laboratory of Road & Traffic Engineering of the Ministry of Education, Tongji University, Shanghai 201804, China

**Keywords:** crash severity, weather condition, generalized ordered logit model, spatial correlation, conditional autoregressive prior, Bayesian inference

## Abstract

This study presents an empirical investigation of the impacts of real-time weather conditions on the freeway crash severity. A Bayesian spatial generalized ordered logit model was developed for modeling the crash severity using the hourly wind speed, air temperature, precipitation, visibility, and humidity, as well as other observed factors. A total of 1424 crash records from Kaiyang Freeway, China in 2014 and 2015 were collected for the investigation. The proposed model can simultaneously accommodate the ordered nature in severity levels and spatial correlation across adjacent crashes. Its strength is demonstrated by the existence of significant spatial correlation and its better model fit and more reasonable estimation results than the counterparts of a generalized ordered logit model. The estimation results show that an increase in the precipitation is associated with decreases in the probabilities of light and severe crashes, and an increase in the probability of medium crashes. Additionally, driver type, vehicle type, vehicle registered province, crash time, crash type, response time of emergency medical service, and horizontal curvature and vertical grade of the crash location, were also found to have significant effects on the crash severity. To alleviate the severity levels of crashes on rainy days, some engineering countermeasures are suggested, in addition to the implemented strategies.

## 1. Introduction

Weather conditions have been found to affect traffic crash risk and severity (e.g., [1,2,3,4,5]). Especially, adverse weather conditions (e.g., typhoon, rainstorm, and heavy fog) may result in severe crashes on rural freeways, which are characterized with a high vehicle speed and a large proportion of heavy vehicles [6]. Quantifying the effects of weather conditions on the crash severity has a potential to provide directions for developing countermeasures and policies aimed at decreasing the amount of property damage and mitigating the level of injury severity sustained by involved road users, and thus improving the safety performance of freeways. 

However, in most of the previous crash severity analyses [6,7,8], researchers collected weather information from historical crash reports, where police officers recorded the weather information based on their subjective judgements at crash scenes or even their memories when they were back at their offices [8]. The vague information on weather conditions may lead to a biased estimation of their effects on crash severity. A better alternative is collecting real-time data on weather conditions from proximate weather observation stations where precise information on wind speed, air temperature, precipitation, visibility, and humidity is usually measured continuously by specific sensors and recorded at small intervals (e.g., 2 or 15 min) [9,10]. Incorporating them into crash severity models is expected to uncover a more explicit relationship between crash severity and weather conditions, as well as the attributes related to drivers, vehicles, roadways, emergency medical service (EMS), and crash configuration.

A number of previous studies have examined the impacts of real-time weather conditions on crash injury severity [8,9,11]. However, these studies are specific to certain crash types. Specifically, Jung, Qin, and Noyce [11] used sequential logistic models to assess the effects of wind speed, temperature, rainfall intensity, and water film depth on the injury severity of single-vehicle and multi-vehicle crashes respectively in rainy weather. They found that wind speed has significant effects on the severity of both single-vehicle and multi-vehicle crashes and rainfall intensity has a significant effect on single-vehicle crashes only. Naik et al. [9] investigated the relationship between the injury severity of single-vehicle truck crashes and real-time weather conditions using mixed ordered and unordered logit regressions. The results showed that more severe injuries in single-vehicle truck crashes are associated with higher wind speed and air temperature, heavier precipitation, and lower humidity. Recently, Zhai et al. [8] developed a mixed logit model to analyze the impacts of weather conditions on pedestrian crash severity and found that higher air temperature and presence of rainfall were linked to a higher level of pedestrian injuries. 

To the best of our knowledge, there is only one reported study [12] that focused on the effects of real-time weather conditions on the injury severity of freeway crashes (regardless of crash types). The importance of explanatory variables was estimated using the random forest method and temperature was identified as the only important weather factor. Nonetheless, there are several limitations of the research with respect to generalization and methodology. First, only two injury levels (no-injury versus injury) were classified, which may result in inadequate use of crash severity data and cannot provide a thorough understanding of the effects of significant factors on the likelihood of certain specific injury levels (e.g., fatality). Second, the factors related to drivers, vehicles, EMS, and crash configuration were not considered in the analysis. Third, the employed support vector machine, and fixed and mixed logit models cannot account for the spatial correlation among adjacent crashes, the significance of which has been demonstrated in extensive previous studies on modeling crash frequency/rate [13,14,15,16,17] and severity [18,19,20,21]. Using a more rigorous modeling scheme for the analysis of crash severity in a more comprehensive metric with more external factors controlled is beneficial to improve the accuracy of the estimated effects of real-time weather conditions on the adverse outcomes of freeway crashes.

Methodologically, the substantial progress in analytical methods over the years has enabled a more precise determination of the influence of risk factors on crash severity. A wide range of sophisticated methods have been developed by accommodating the fundamental characteristics of crash severity data, including ordered nature [22,23], underreporting [24], endogeneity [25], within-crash correlation [26,27], spatial and temporal correlation [19,20,28,29], unobserved heterogeneity [30,31], etc. Please refer to [32,33] for a comprehensive introduction and assessment on the methodological alternatives. More recently, a Bayesian spatial generalized ordered logit model proposed by Zeng et al. [6] is one of the state-of-the-art methods for modeling crash severity. The model is able to account for the ordered nature and spatial correlation simultaneously. The thresholds are allowed to vary with the observed explanatory variables, which can remove the restrictions imposed by the fixed thresholds in standard ordered response models [34]. Moreover, the conditional autoregressive (CAR) priors incorporated can accommodate not only the spatial correlation across crashes but also the unobserved heterogeneity [19].

In the current research, the Bayesian spatial generalized ordered logit model was developed to investigate the impacts of real-time weather conditions on freeway crash severity. A comprehensive crash dataset collected from Kaiyang Freeway in Guangdong Province, China in 2014 and 2015 was used for the empirical investigation, where crash severity is categorized by Chinese police administration according to an integrative assessment of the adverse crash outcomes, i.e., the number of people injured at various degrees (e.g., slight and serious injury, and fatality) and the amount of property damage. To demonstrate the superiority of the proposed spatial model, it is compared with a generalized ordered logit model in terms of model fit and parameter estimates.

The remainder of this paper is organized as follows: In Section 2, we introduce the collected freeway crash dataset for the analysis. In Section 3, we specify the formulations of the traditional and spatial generalized ordered logit models, the criteria for model fit comparison, and the calculation of the marginal effects of risk factors. Section 4 presents the Bayesian estimation process of the models and analyzes the results of the model comparison and estimation. In Section 5, some remarkable conclusions are drawn and several directions for future research are provided.

## 2. Data Assembly

A comprehensive dataset from the Kaiyang Freeway in 2014 and 2015 was used in the current research. It was assembled with information from three different resources on crash data, roadway inventory, and real-time weather conditions, respectively. 

### 2.1. Crash Data

We obtain the freeway crash data from the Highway Maintenance and Administration Management System, which is maintained by Guangdong Transportation Group (Guangzhou, China). In the system, crash severity is classified into four ordered levels according to the criteria defined by the Ministry of Public Security in China. Specifically,

1. a “light crash” refers to one resulting in a property damage value of no more than 1000 CNY, or no more than two people slightly injured;
2. a “medium crash” refers to one resulting in a property damage value between 1000 and 30,000 CNY, or more than two people slightly injured, or one or two people severely injured;
3. a “severe crash” refers to one resulting in a property damage value between 30,000 and 60,000 CNY, or three to ten people severely injured, or one or two fatalities; and
4. a “very severe crash” refers to one resulting in a property damage value of over 60,000 CNY, or more than ten people severely injured, or more than eight people severely injured and one fatality, or more than five people severely injured and two fatalities, or no less than three fatalities.

Among all the 1424 freeway crashes reported in the two years, there were 756 light crashes (53.1%), 621 medium crashes (43.6%), 45 severe crashes (3.2%), and only two very severe crashes (0.1%). Due to the rareness of very severe crashes, they were combined with severe crashes, to constitute the highest level (termed as “severe crash” in the rest of the paper) of crash severity in the research. 

Some important features of driver, vehicle, EMS, and crash configuration are also recorded in the system, including: whether the involved driver(s) were professional (i.e., those taking vehicle driving as their jobs) or not, the involved vehicles’ types and license numbers, the EMS response time, and the crash type, time and location (recorded as kilometer markers on the freeway). 

### 2.2. Roadway Inventory

We extracted more detailed roadway characteristics of crash locations from the freeway geometric profile provided by Guangdong Province Communication Planning and Design Institute Co., Ltd. (Guangzhou, China). These roadway characteristics include horizontal curvature, vertical grade, and whether the crash location is on a bridge or near a ramp. To explore the spatial correlation in the crashes, Kaiyang Freeway was split into 154 segments according to the homogeneity in horizontal and vertical alignments, which is consistent to the freeway segmentation in our previous studies on freeway crash analysis [35,36].

### 2.3. Real-Time Weather Conditions

The weather data from three county-level weather stations along the freeway were drawn from the Meteorological Information Management System (MIMS) maintained by the Guangdong Climate Center. In the MIMS, weather indexes, which include wind speed, air temperature, precipitation, visibility, and humidity, are recorded hourly. The crashes are assigned to the nearest weather station in accordance with their crash locations [9,12]. For each crash, the weather indexes observed at the assigned weather station during the hour of the crash time were used to reveal the real-time weather conditions.

Table 1 shows the definitions and descriptive statistics of the explanatory variables for the empirical analysis. 

## 3. Methodology

In this section, the structures of generalized ordered logit model and spatial generalized ordered logit model for analyzing crash severity are presented first (Section 3.1). We then introduce two criteria for assessing the performance of the two models in the context of Bayesian inference (Section 3.2). Finally, the method for calculating the marginal effects of explanatory variables is described (Section 3.3). 

### 3.1. Model Specification

#### 3.1.1. Generalized Ordered Logit Model

Ordered nature is an important characteristic of crash-severity data [32,33]. The generalized ordered logit model can accommodate the characteristic appropriately, without suffering from inconsistent estimations caused by fixed thresholds. Specifically, the severity level, $y_{i},$ of crash i is formulated as follows:

(1) $$y_{i}=\{\begin{array}{cc} 1, & z_{i}\leq \mu_{i,1}\\ 2, & \mu_{i,1}<z_{i}\leq \mu_{i,2}\\ 3, & z_{i}>\mu_{i,2} \end{array},$$

where 1, 2, 3 denotes the crash severity levels categorized above, i.e., light crash, medium crash, and severe crash, respectively. $z_{i}$ is a latent variable indicating the latent severity propensity of crash i and is assumed to be a linear function of the explanatory variables (including a constant element) $X_{i}$:

(2) $$z_{i}=\beta X_{i}+\varepsilon_{i},$$

where β is a vector of estimable parameters corresponding to $X_{i}$, and $\varepsilon_{i}$ is a residual term which is assumed to follow a logistic distribution.

The thresholds $\mu_{i,1}$ and $\mu_{i,2}$ in Equation (1) represent the boundaries between the ordered severity levels for crash i. To allow flexibility in measuring the effects of explanatory variables, the relationship between the thresholds is defined as follows:

(3) $$\mu_{i,2}=\mu_{i,1}+\exp\left(\alpha Z_{i}\right),$$

where $Z_{i}$ is a vector of explanatory variables (also including a constant element) and α is the corresponding parameter vector. For the uniqueness of identification, and without loss of generality, the threshold between light and medium crash levels, $\mu_{i,1}$, is fixed to zero for all crashes.

As the residual term $\varepsilon_{i}$ is logistically distributed, the cumulative probability for crash i to present a severity level up to j (=1, 2, 3), $P_{i,j}$, can be calculated as:

(4) $$P_{i,1}=\frac{\exp\left(\mu_{i,1}-\beta X_{i}\right)}{1+\exp\left(\mu_{i,1}-\beta X_{i}\right)}=\frac{\exp\left(-\beta X_{i}\right)}{1+\exp\left(-\beta X_{i}\right)},$$

(5) $$P_{i,2}=\frac{\exp\left(\mu_{i,2}-\beta X_{i}\right)}{1+\exp\left(\mu_{i,2}-\beta X_{i}\right)}=\frac{\exp\left[\exp\left(\alpha Z_{i}\right)-\beta X_{i}\right]}{1+\exp\left[\exp\left(\alpha Z_{i}\right)-\beta X_{i}\right]},$$

(6) $$P_{i,3}=1.$$

Consequently, the probability for crash i resulting in the jth level of severity, $p_{i,j}$, is calculated as:

(7) $$p_{i,1}=P_{i,1}=\frac{\exp\left(-\beta X_{i}\right)}{1+\exp\left(-\beta X_{i}\right)},$$

(8) $$p_{i,2}=P_{i,2}-P_{i,1}=\frac{\exp\left(-\beta X_{i}\right)\left[\exp\left(\exp\left(\alpha Z_{i}\right)\right)-1\right]}{\left[1+\exp\left(-\beta X_{i}\right)\right]\left[1+\exp\left(\exp\left(\alpha Z_{i}\right)-\beta X_{i}\right)\right]},$$

(9) $$p_{i,3}=1-P_{i,2}=\frac{1}{1+\exp\left[\exp\left(\alpha Z_{i}\right)-\beta X_{i}\right]}.$$

#### 3.1.2. Spatial Generalized Ordered Logit Model

Spatial correlation is also a fundamental characteristic of crash-severity data [32,33]. The spatial generalized ordered logit model is developed by accounting for the ordered nature and spatial correlation simultaneously [6]. Specifically, a residual term $\varphi_{m}$ with CAR priors is added into the formulation of the latent severity propensity, that is,

(10) $$z_{i}=\beta X_{i}+\varepsilon_{i}+\varphi_{m},$$

(11) $$\varphi_{m}\sim N\left(\frac{\sum_{n\neq m}\omega_{m,n}\varphi_{n}}{\sum_{n\neq m}\omega_{m,n}},\frac{1}{\tau_{\varphi }\sum_{n\neq m}\omega_{m,n}}\;\right),$$

where the $\varphi_{m}$ captures the spatial effects of crashes (including crash i) occurring on roadway segment m. The $\omega_{m,n}$ is the adjacency weight for roadway segments m and n in the proximity matrix. The most extensively used structure [6,20,24], a binary first-order neighbor, was employed to define the proximity matrix in the current research. Specifically, $\omega_{m,n}=1$, if segments m and n are connected; $\omega_{m,n}=0$, otherwise. The $\tau_{\varphi }(>0)$ is the precision parameter of the spatial correlation term.

Thus, the probability for crash i to exhibit the jth severity level is formulated as:

(12) $$p_{i,1}=\frac{\exp\left(-\beta X_{i}-\varphi_{m}\right)}{1+\exp\left(-\beta X_{i}-\varphi_{m}\right)},$$

(13) $$p_{i,2}=\frac{\exp\left(-\beta X_{i}-\varphi_{m}\right)\left[\exp\left(\exp\left(\alpha Z_{i}\right)\right)-1\right]}{\left[1+\exp\left(-\beta X_{i}-\varphi_{m}\right)\right]\left[1+\exp\left(\exp\left(\alpha Z_{i}\right)-\beta X_{i}-\varphi_{m}\right)\right]},$$

(14) $$p_{i,3}=\frac{1}{1+\exp\left[\exp\left(\alpha Z_{i}\right)-\beta X_{i}-\varphi_{m}\right]}.$$

### 3.2. Assessment Criteria

The performances of the above models were compared via the deviance information criterion (DIC) and classification accuracy. As a Bayesian generalization of Akaike information criterion (AIC) and Bayes information criterion (BIC), the DIC provides a combined measure of model fit and complexity. Specifically, it is defined as [37]:

(15) $$\mathrm{DIC}=\bar{D}+p_{D},$$

where $\bar{D}$ is the posterior mean deviance, which can be taken as a Bayesian measure of model fit, and $p_{D}$ is the effective number of model parameters that can be used to measure model complexity. The lower the DIC value, the better the overall model performance. Empirically, over 10 differences can rule out the model with a higher DIC [38].

The classification accuracy for the whole dataset is calculated as [6]:

(16) $$\mathrm{CA}=\frac{\sum_{y_{i}={\bar{y}}_{i}}y_{i}/y_{i}}{\sum_{i}y_{i}/y_{i}},$$

where $\bar{y}$ is the predicted severity level of crash i.

### 3.3. Marginal Effects

Understanding the impacts of explanatory variables within the framework of generalized ordered response modeling is not straightforward. The regression coefficients β and α in the proposed model do not directly provide the magnitude of the effects of a certain explanatory variable on the likelihood of each severity level. For this purpose, the marginal effects of significant variables in the spatial model are calculated. Specifically, the marginal effect of a continuous variable x on $p_{i,j}$ is computed by taking the first-order derivative with respect to x [6,39]:

(17) $$\frac{\partial p_{i,1}}{\partial x}=\beta^{x}p_{i,1}\left(p_{i,1}-1\right),$$

(18) $$\frac{\partial p_{i,2}}{\partial x}=\alpha^{x}\mu_{i}p_{i,3}\left(1-p_{i,3}\right)+\beta^{x}p_{i,2}\left(p_{i,1}-p_{i,3}\right),$$

(19) $$\frac{\partial p_{i,3}}{\partial x}=\left(\beta^{x}-\alpha^{x}\mu_{i}\right)p_{i,3}\left(1-p_{i,3}\right),$$

where $\beta^{x}$ and $\alpha^{x}$ are the coefficient estimates associated with variable x in the functions of latent propensity, $z_{i},$ and threshold, $\mu_{i}$, respectively.

For an indicator (binary) variable, x, its marginal effect on $p_{i,j}$ is calculated as the difference in the estimated probabilities with it varying from zero to one (Δx=1):

(20) $$\frac{\Delta p_{i,1}}{\Delta x}=\frac{\exp\left(-\tilde{\beta }{\tilde{X}}_{i}-\varphi_{m}\right)\left[\exp\left(-\beta^{x}\right)-1\right]}{\left[1+\exp\left(-\tilde{\beta }{\tilde{X}}_{i}-\varphi_{m}\right)\right]\left[1+\exp\left(-\tilde{\beta }{\tilde{X}}_{i}-\beta^{x}-\varphi_{m}\right)\right]},$$

(21) $$\frac{\Delta p_{i,2}}{\Delta x}=\frac{\exp\left(-\tilde{\beta }{\tilde{X}}_{i}-\beta^{x}-\varphi_{m}\right)\left\{\exp\left[\exp\left(\tilde{\alpha }{\tilde{Z}}_{i}+\alpha^{x}\right)\right]-1\right\}}{\left\{1+\exp\left[\exp\left(\tilde{\alpha }{\tilde{Z}}_{i}+\alpha^{x}\right)-\tilde{\beta }{\tilde{X}}_{i}-\beta^{x}-\varphi_{m}\right]\right\}\left\{1+\exp\left[-\tilde{\beta }{\tilde{X}}_{i}-\beta^{x}-\varphi_{m}\right]\right\}}\;-\frac{\exp\left(-\tilde{\beta }{\tilde{X}}_{i}-\varphi_{m}\right)\left\{\exp\left[\exp\left(\tilde{\alpha }{\tilde{Z}}_{i}\right)\right]-1\right\}}{\left\{1+\exp\left[\exp\left(\tilde{\alpha }{\tilde{Z}}_{i}\right)-\tilde{\beta }{\tilde{X}}_{i}-\varphi_{m}\right]\right\}\left\{1+\exp\left[-\tilde{\beta }{\tilde{X}}_{i}-\varphi_{m}\right]\right\}}$$

(22) $$\frac{\Delta p_{i,3}}{\Delta x}=\frac{\exp\left(-\tilde{\beta }{\tilde{X}}_{i}-\varphi_{m}\right)\left\{\exp\left[\exp\left(\tilde{\alpha }{\tilde{Z}}_{i}\right)\right]-\exp\left[\exp\left(\tilde{\alpha }{\tilde{Z}}_{i}+\alpha^{x}\right)-\beta^{x}\right]\right\}}{\left\{1+\exp\left[\exp\left(\tilde{\alpha }{\tilde{Z}}_{i}\right)-\tilde{\beta }{\tilde{X}}_{i}-\varphi_{m}\right]\right\}\left\{1+\exp\left[\exp\left(\tilde{\alpha }{\tilde{Z}}_{i}+\alpha^{x}\right)-\tilde{\beta }{\tilde{X}}_{i}-\beta^{x}-\varphi_{m}\right]\right\}},$$

where ${\tilde{X}}_{i}$ and ${\tilde{Z}}_{i}$ are the vectors $X_{i}$ and $Z_{i}$ less element x, respectively, and $\tilde{\beta }$ and $\tilde{\alpha }$ are the corresponding parameter vectors (i.e., β less $\beta^{x}$ and α less $\alpha^{x}$, respectively). 

The calculations of the marginal effects (no matter for continuous variables or indicator variables) are specific to a certain crash. To represent the whole dataset, the average marginal effects for all observations are computed and reported.

## 4. Results and Discussion

### 4.1. Model Estimation

Since the traditional maximum-likelihood method is not applicable to the models with CAR Gaussian priors [40], the parameters in the models were calibrated by Bayesian method which can be easily conducted via programming in WinBUGS [41]. To obtain the Bayesian estimates, specification of the prior distribution of each parameter in the models is required. In the absence of sufficient knowledge, noninformative (vague) prior distributions were used for the parameters. To be specific, a diffused normal distribution, $Normal\left(0,\;10^{4}\right)$, was used as the priors of the coefficients in β and α. A diffused gamma distribution, gamma(0.01, 0.01), was used as the priors of the spatial precision parameter, $\tau_{\varphi }$. The CAR priors were specified by the function *car.normal* in WinBUGS [40]. For each model, a chain of 60,000 Markov chain Monte Carlo (MCMC) simulation iterations was run, with the first 50,000 iterations acting as a burn-in. The MCMC trace plots for the model parameters were inspected visually to ensure the simulations converge. In addition, we monitored the ratios between the Monte Carlo simulation errors and the respective estimates’ standard deviations to ensure that they were less than 0.05 (a rule-of-thumb threshold). The estimation and assessment results for the traditional and spatial generalized ordered logit models are summarized in Table 2 and Table 3, where only the factors that have statistically significant (at least at 90% credibility level) effects on the latent propensity or threshold are included. 

### 4.2. Model Comparison

Comparing the results in Table 2 and Table 3, one can observe that the spatial generalized ordered logit model yields a lower $\bar{D}$ value, which indicates its better fitting with the crash data. The outperformance of the spatial model in goodness-of-fit is further confirmed by its relatively higher classification accuracy (76% for the spatial model versus 75% for the traditional model). The results are reasonable, because a number of previous studies [6,19,20] have demonstrated that capturing spatial effects via CAR priors can significantly reduce model misspecification. While the generalized ordered logit model is more parsimonious (as suggested by the lower $p_{D}$ value), the DIC value of the spatial model is 13 points lower than that of the traditional one, which implies the better overall performance of the proposed spatial model.

In addition, the standard deviation of the spatial term, sd(φ), was estimated. Its posterior mean equals 0.56 and the 95% Bayesian credible interval is (0.32, 0.84), which manifest that there are significant spatial correlations among crashes occurring on adjacent freeway segments. The spatial correlations may be attributed to some omitted factors (e.g., terrain feature and lighting condition) shared by adjacent crashes.

Further comparison between the two models shows that there are certain discrepancies in the identified significant factors of crash severity. For example, horizontal curvature was found to be positively associated with the latent severity propensity only in the spatial model, while wind speed was found to be negatively associated with the latent severity propensity only in the generalized ordered logit model. The results also imply that the spatial model is more consistent with the findings in the literature than the traditional one. Note that many studies have reported that: (i) crashes occurring on segments with higher smaller horizontal curve radius tend to be more severe [7,42]; and (ii) stronger wind increases the likelihood of severe crashes [43].

### 4.3. Parameter and Marginal Effect Interpretation

The marginal effects of significant factors on the probability of each crash severity level were calculated for the spatial model via the method in Section 3.3. The results are shown in Table 4. This research mainly aims to assess the impacts of real-time weather conditions on freeway crash severity. Therefore, we interpret the estimated regression coefficients and marginal effects of real-time weather index(es) first (Section 4.3.1) and then those of other significant variables (Section 4.3.2). 

#### 4.3.1. Real-Time Weather Conditions

According to the results in Table 2 and Table 3, precipitation has significantly positive effects on both the latent severity propensity and the threshold between medium and severe crashes. Specifically, a one-millimeter increase in precipitation during the hour of the crash time tends to result in the likelihood of light and severe crashes decreasing by 0.6% and 1.0%, respectively, and the likelihood of medium crashes increasing by 1.6%. The decreased likelihood of light crashes is anticipated, because precipitation makes the roadway surface wet or even slippery, thereby reducing skidding resistance [4,44]. The reduced skidding resistance increases the difficulties in manipulating vehicles, which could increase drivers’ mental effort and thereby adversely influence driving behavior by occupying limited cognitive resources and interfering with information processing. Once an emergency occurs, drivers may need more time to perceive its existence and take proper actions to reduce the severity of an oncoming crash. Moreover, the reduced skidding resistance also increases stopping distance.

**Figure 1:**
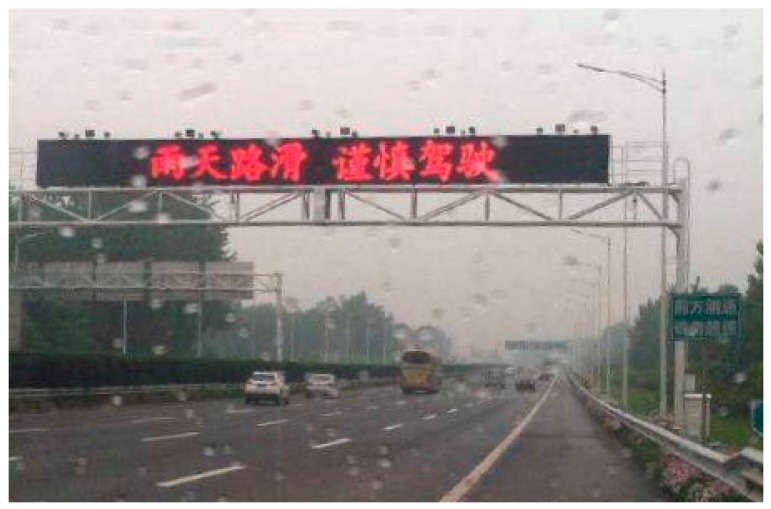
Variable message sign.

While it is somewhat counterintuitive, the decreased probability of severe crashes in heavy rain conforms to many existing findings of the effects of the wet road surface on crash severity [7,45,46]. They argued that it could be illustrated by the risk compensation theory, which indicates that drivers tend to adjust their driving behavior (e.g., driving more carefully and at a lower speed) in adverse driving conditions (e.g., heavy rain or wet road surface). In practice, some transportation engineering and management strategies are implemented to enhance freeway safety. For example, the variable message signs (as shown in Figure 1) deployed along the freeway would be activated to alert drivers to be cautious on rainy days. The transportation management agency usually sets a more intensive police patrol schedule in seasons with high precipitation.

Furthermore, it is worth noting that precipitation impacts the likelihood of light and severe crashes in the same direction, which cannot be formulated in standard ordered response models. The fixed thresholds in standard ordered response models limit that the marginal effects of a certain factor on the probabilities of the lowest and highest severity levels always have different signs [34]. The results justify the necessity of modeling crash severity under a generalized response framework.

#### 4.3.2. Other Significant Variables

Regarding other significant variables, professional drivers were associated with a higher latent severity propensity and a higher threshold between medium and severe crashes, which indicate that the probability of light crashes is expected to decrease by 32.8% and that the probabilities of medium and severe crashes are expected to increase by 27.2% and 5.6%, respectively, when there are professional drivers involved in the crash. The result is generally consistent with the finding of our previous research [6]. In the collected crash data, most professional drivers operated intercity buses. Because of the long driving hours, they are more likely to experience fatigue driving, which may increase the likelihood of severe crashes [47]. 

The positive signs of the coefficients for coaches and other vehicles on the latent severity propensity imply that these two types of vehicles are more likely to be involved in severe crashes. The estimated marginal effects showed that, when a coach is involved, the likelihood of a severe crash will increase by 1.8%; while when another type of vehicle (e.g., a vehicle with trailer) is involved, the counterpart will increase by 2.7%. The results are reasonable, because coaches and other types of vehicles possess stronger crash aggressivity, compared to automobiles [27]. The stronger crash aggressivity means that greater hazards would be imposed on the vehicle(s) colliding with them [48].

“Non-local vehicle” has a negative effect on the latent severity propensity, which indicates that non-local vehicles are more likely to be involved in severe crashes. Specifically, when at least one non-local vehicle is involved, the probabilities that the crash severity is medium and severe will increase by 3.4% and 0.9%, respectively. The result is generally consistent with engineering intuition: the drivers of non-local vehicles may be unfamiliar with the roadway and weather conditions. As a consequence, they may need more time to perceive and comprehend the driving environment. Thus, less time is left for them to slow down or perform other actions that could alleviate the adverse outcomes of an upcoming crash.

For the crash time of day, afternoon is linked to a higher threshold between medium and severe crashes, while evening is linked to a lower latent severity propensity. The results of marginal effects indicate that the probabilities of severe crashes in afternoon and evening decrease by 3.3% and 1.3%, respectively, compared to their counterparts before dawn (the reference category). Probably due to the light traffic, speeding is more likely to occur before dawn [26]. Moreover, human circadian rhythmicity may lead to more frequent fatigue/sleep-deprived driving during this period [49]. Speeding and fatigue driving are major causes of severe crashes in China [6]. Moreover, drivers’ vision is better in the afternoon than before dawn, which reserves more time for drivers to recognize and respond to potential dangers [7].

With respect to roadway characteristics, horizontal curvature has a positive effect on the latent severity propensity, which implies that the probabilities of medium and severe crashes will increase by 1.5% and 0.4% respectively, for a 10^−1^ km increase in horizontal curvature. A greater curvature (i.e., a smaller curve radius) makes for stronger centrifugal forces on vehicles negotiating the curve and brings about the harsher transition between tangent sections [50], which may lead to a reduction in vehicle control. Zegeer et al. [42] claimed that more head-on crashes, fixed object crashes, and rollover crashes tend to occur on horizontal curves. These crashes usually result in great casualties. “Vertical grade” was found to be negatively associated with the threshold between medium and severe crashes. Specifically, a 1% increase in the vertical grade is expected to result in a 2.2% increase in the probability of severe crashes. This finding may be attributed to a shorter sight distance rendered by a steeper grade, which reduces the time available for drivers to react properly to potential hazards [7,46].

By providing first aid treatments and transportation to hospitals, EMS is a crucial post-crash countermeasure for mitigating the injuries sustained by the occupants involved in traffic crashes. It is anticipated that the EMS response time has a positive impact on the latent severity propensity. Its estimated marginal effects reveal that an increase of one minute in EMS response time will increase the likelihood of medium and severe crashes by 0.4% and 0.1%, respectively, which is in line with the findings in many previous studies [6,51,52].

With regard to crash type, the results in Table 3 indicate that both rear-end and angle crashes are linked to a reduction in the latent severity propensity and a reduction in the threshold between medium and severe crashes, as compared against single-vehicle crashes (the reference category). Specifically, the probability of light crashes increases by 47.5% and 36.1% for rear-end and angle crashes, respectively. The probability of severe crashes decreases by 0.2% for rear-end crashes but increases by 2.7% for angle crashes. Similar findings can be found in [27,48], which concluded that rear-end crashes are one of the least severe crash types.

## 5. Conclusions

This paper empirically investigated the impacts of real-time weather conditions on freeway crash severity using a two-year crash dataset collected from Kaiyang Freeway in China, where the information on hourly wind speed, air temperature, precipitation, visibility, and humidity were derived from three adjacent weather stations. A state-of-the-art method, the Bayesian spatial generalized ordered logit model, was used for the empirical analysis, to link the observed crash severity to real-time weather conditions and factors related to drivers, vehicles, roadways, EMS, and crash configuration.

The results indicate that heavier precipitation during the hour of crash occurrence decreases the probabilities of light and severe crashes but increases the probability of medium crashes. The decreased probability of severe crashes may imply the effects of some implemented strategies for transportation safety management, including variable message signs and police patrol schedules. Nonetheless, some other strategies may further improve freeway safety performance on rainy days. For example, variable speed limits have the potential to reduce crash risk and severity in rainy weather, by continually regulating travel speed based on real-time traffic and weather conditions [53]. The advanced driver-assistance system and emerging connected and autonomous vehicles constantly detect potential dangers and facilitate drivers to make proper response decisions, which is especially helpful in inclement weather.

The results also suggest that: (1) professional drivers, coaches, other vehicles (especially those with trailers), and non-local vehicles are more likely to be involved in severe crashes; (2) severe crashes tend to occur on freeway segments with small horizontal curve radius and high vertical gradient before dawn; (3) rapid response of EMS can significantly decrease crash severity; (4) rear-end crashes usually result in less severe outcomes than single-vehicle and angle crashes; (5) significant spatial correlation exists across the severities of adjacent crashes.

A limitation of the current research is that the weather information was recorded by hour. Higher-resolution weather data (e.g., at 1-, 5-, or 10-min intervals) may provide a more precise assessment of their effects on crash severity. Methodology-wise, it is of interest to further account for the heterogeneous effects of the observed factors in the proposed model by using methods such as random-parameters [30], although it may significantly increase the complexity of model structure and the time-consumed in model estimation. In addition, more field data are required to demonstrate the random-parameters model.

## Figures and Tables

**Table 1:** Descriptive statistics of explanatory variables for analyzing freeway crash severity.

Covariates	Description	Mean	SD
Professional driver	All drivers involved are non-professional = 0; otherwise = 1	0.039	0.193
EMS response time	Duration between crash reporting and the arrival of EMS (min)	19.4	16.6
Day of week	Crash occurred on a weekend = 1; otherwise = 0	0.345	0.476
VEHICLE TYPE			
Passenger car *	All vehicles involved are passenger cars = 1; otherwise = 0	0.579	0.494
Coach	At least one coach was involved = 1; otherwise = 0	0.064	0.245
Truck	At least one truck was involved = 1; otherwise = 0	0.313	0.464
Other vehicle	At least one other vehicle (e.g., a vehicle with trailer) was involved = 1; otherwise = 0	0.099	0.299
Non-local vehicle	All vehicles involved were registered in Guangdong Province (local vehicles) = 0; otherwise (at least one non-local vehicle was involved) = 1	0.284	0.451
CRASH TYPE			
Single-vehicle crash *	The crash involved only one vehicle = 1; otherwise = 0	0.454	0.498
Rear-end crash	The crash is a rear-end one = 1; otherwise = 0	0.383	0.486
Angle crash	The crash is an angle one where the directions of involved vehicles are not parallel = 1; otherwise = 0	0.162	0.368
TIME OF DAY			
Before dawn *	Crash occurred during 12 a.m. to 6 a.m. = 1; otherwise = 0	0.184	0.387
Morning	Crash occurred during 6 a.m. to 12 p.m. = 1; otherwise = 0	0.222	0.416
Afternoon	Crash occurred during 12 p.m. to 6 p.m. = 1; otherwise = 0	0.372	0.483
Evening	Crash occurred during 6 p.m. to 12 a.m. = 1; otherwise = 0	0.222	0.416
ROADWAY GEOMETRY			
Horizontal curvature	The horizontal curvature of the freeway segment where the crash occurred (0.1 km^−1^)	1.84	1.23
Vertical grade	The grade of the freeway segment where the crash occurred (%)	0.710	0.592
Bridge	Crash occurred on a bridge = 1; otherwise = 0	0.537	0.499
Ramp	Crash occurred in the proximity of a ramp = 1; otherwise = 0	0.244	0.430
REAL-TIME WEATHER CONDITION			
Wind speed	Wind speed during the hour of crash time (m/s)	3.83	2.06
Temperature	Air temperature during the hour of crash time (°C)	23.7	6.08
Precipitation	Precipitation during the hour of crash time (mm)	0.769	3.43
Visibility	Visibility during the hour of crash time (km)	18.0	18.7
Humidity	Humidity during the hour of crash time (%)	81.3	15.5

* The reference category. EMS: emergency medical service.

**Table 2:** Estimation and assessment results for the generalized ordered logit model.

Variable	Latent Severity Propensity			Threshold between Median and Severe Crash Levels		
	Mean	90% BCI ^a^	95% BCI	Mean	90% BCI	95% BCI
Constant	0.64	(0.01, 1.29)	(−0.09, 1.39)	1.75	(1.32, 2.14)	(1.22, 2.21)
Precipitation	0.06	(0.02, 0.09)	(0.01, 0.10)	0.12	(0.02, 0.24)	(0.01, 0.26)
Rear-end crash	−2.47	(−2.75, −2.20)	(−2.81, −2.12)	−0.80	(−1.03, −0.55)	(−1.08, −0.50)
Angle crash	−2.10	(−2.42, −1.78)	(−2.49, −1.73)	−0.83	(−1.11, −0.56)	(−1.16, −0.51)
Professional driver	2.11	(1.36, 2.97)	(1.2, 3.21)	0.32	(0.05, 0.60)	(0.001, 0.66)
Coach	0.59	(0.16, 1.01)	(0.06, 1.09)	—	—	—
Other vehicle	0.83	(0.44, 1.21)	(0.37, 1.29)	—	—	—
EMS response time	0.027	(0.019, 0.035)	(0.018, 0.036)	—	—	—
Wind speed	−0.07	(−0.13, −0.01)	(−0.14, 0.002)	—	—	—
Vertical grade	—	—	—	−0.23	(−0.37, −0.11)	(−0.40, −0.08)
Afternoon	—	—	—	0.42	(0.18, 0.68)	(0.13, 0.73)
Evening	−0.45	(−0.79, −0.10)	(−0.85, −0.03)	—	—	—
$\bar{D}$	1720	—	—	—	—	—
$p_{D}$	41	—	—	—	—	—
DIC	1761	—	—	—	—	—
CA	75%	—	—	—	—	—

^a^ BIC: Bayesian credible interval. $\bar{D}$: posterior mean deviance; $p_{D}$: effective number of model parameters; DIC: deviance information criterion; CA: classification accuracy.

**Table 3:** Estimation and assessment results for the spatial generalized ordered logit model.

Variable	Latent Severity Propensity			Threshold between Median and Severe Crash Levels		
	Mean	90% BCI ^a^	95% BCI	Mean	90% BCI	95% BCI
Constant	2.7	(1.35, 3.99)	(1.20, 4.18)	1.73	(1.30, 2.13)	(1.23, 2.21)
Precipitation	0.04	(0.004, 0.08)	(−0.01, 0.09)	0.13	(0.03, 0.25)	(0.02, 0.28)
Rear-end crash	−2.53	(−2.82, −2.24)	(−2.88, −2.19)	−0.80	(−1.04, −0.57)	(−1.09, −0.53)
Angle crash	−1.84	(−2.19, −1.50)	(−2.27, −1.44)	−0.81	(−1.09, −0.55)	(−1.15, −0.50)
Professional driver	2.23	(1.45, 3.11)	(1.33, 2.29)	0.33	(0.06, 0.60)	(0.004, 0.65)
Coach	0.48	(0.23, 0.93)	(−0.05, 1.00)	—	—	—
Other vehicle	0.71	(0.30, 1.11)	(0.23, 1.18)	—	—	—
Non-local vehicle	0.28	(0.01, 0.57)	(−0.06, 0.62)	—	—	—
EMS response time	0.03	(0.025, 0.043)	(0.024, 0.045)	—	—	—
Horizontal curvature	0.13	(0.03, 0.23)	(0.01, 0.25)	—	—	—
Vertical grade	—	—	—	−0.24	(−0.38, −0.11)	(−0.41, −0.07)
Afternoon	—	—	—	0.42	(0.16, 0.68)	(0.11, 0.75)
Evening	−0.43	(−0.81, −0.02)	(−0.89, 0.05)	—	—	—
sd(φ) ^b^	0.54	(0.36, 0.80)	(0.32, 0.84)	—	—	—
$\bar{D}$	1684	—	—	—	—	—
$p_{D}$	64	—	—	—	—	—
DIC	1748	—	—	—	—	—
CA	76%	—	—	—	—	—

^a^ BIC: Bayesian credible interval; sd(φ) ^b^ denotes the standard deviation of the spatial term.

**Table 4:** Marginal effects of significant variables in the spatial generalized ordered logit model.

Variable	Light Crashes (%)	Medium Crashes (%)	Severe Crashes (%)
Precipitation	−0.6	1.6	−1.0
Rear-end crash	47.5	−47.3	−0.2
Angle crash	36.1	−38.8	2.7
Professional driver	−32.8	27.2	5.6
Coach	−7.4	5.6	1.8
Other vehicle	−11.0	8.3	2.7
Non-local vehicle	−4.3	3.4	0.9
EMS response time	−0.5	0.4	0.1
Horizontal curvature	−1.9	1.5	0.4
Vertical grade	0.0	−2.2	2.2
Afternoon	0.0	3.3	−3.3
Evening	6.7	−5.4	−1.3
